# Comparative Study on Excretive Characterization of Main Components in Herb Pair Notoginseng-Safflower and Single Herbs by LC–MS/MS

**DOI:** 10.3390/pharmaceutics10040241

**Published:** 2018-11-18

**Authors:** Ying-Yuan Lu, Jin-Yang Song, Yan Li, Yu-Qing Meng, Ming-Bo Zhao, Yong Jiang, Peng-Fei Tu, Xiao-Yu Guo

**Affiliations:** School of Pharmaceutical Sciences, State Key Laboratory of Natural and Biomimetic Drugs, Peking University, Beijing 100191, China; luyingyuan2005@126.com (Y.-Y.L.); songjinyang@bjmu.edu.cn (J.-Y.S.); leeyan@pku.edu.cn (Y.L.); mengyuqing2011@126.com (Y.-Q.M.); zmb@bjmu.edu.cn (M.-B.Z.); yongjiang@bjmu.edu.cn (Y.J.); pengfeitu@bjmu.edu.cn (P.-F.T.)

**Keywords:** notoginseng, safflower, herb pair, LC–MS/MS, urinary excretion, fecal excretion

## Abstract

The herbal medicine combination of notoginseng-safflower has been commonly used clinically for the prevention and treatment of cardiovascular diseases. A reliable liquid chromatography-tandem mass spectrometry (LC–MS/MS) method was developed for simultaneous determination of six bioactive components (hydroxysafflor yellow A, notoginsenoide R1, ginsenoside Rb1, Re, Rd, and Rg1) in rat urine and feces after oral administration of notoginseng total saponins (NS), safflower total flavonoids (SF), and the combination of NS and SF (CNS). The chromatographic separation was achieved on a Waters HSS T3 column under gradient elution with acetonitrile and water containing formic acid as the mobile phase. The calibration curves were linear, with correlation coefficient (*r*) > 0.99 for six components. The intra- and interday precision (RSD) and accuracy (RE) of QC samples were within −14.9% and 14.9%, respectively. The method was successfully applied to study of the urinary and fecal excretion of six bioactive constituents following oral administration of NS, SF, and CNS in rats. Compared to the single herb, the cumulative excretion ratios of six constituents were decreased in the herbal combination. The study indicated that the combination of notoginseng and safflower could reduce the renal and fecal excretion of the major bioactive constituents and promote their absorption in rats.

## 1. Introduction

Traditional Chinese medicine (TCM) has been widely used for the treatment of various diseases in China and other Asian countries for thousands of years, especially for the chronic diseases [1,2,3,4,5]. Many Chinese therapeutic herbs, such as Semen Strychni and Radix Paeoniae Alba, show better pharmacological effects when used in combination than individually. Traditional Chinese herb pairs, which consist of two standard single herbs, are the basic unit in traditional Chinese prescriptions and have the effect of reducing toxicity and increasing efficacy of the herbal medicine [6,7,8].

Notoginseng Radix et Rhizoma (notoginseng) in combination with Carthami Flos (safflower), has been commonly used for the prevention and treatment of cardiovascular diseases [9,10]. Notoginseng is an important Chinese medicinal herb utilized for the treatment of pectoris and coronary artery disease [11,12,13,14]. Notoginseng total saponins (NS), mainly containing notoginsenoside R1, ginsenoside Rg1, Rb1, Re, and Rd, are the most important bioactive ingredients in notoginseng [15]. The dried flowers of safflower have been widely used to treat coronary heart disease, angina pectoris, and hypertension in TCM prescriptions, which mainly contain flavonoids in the extract of safflower (safflower total flavonoids, SF) [16,17,18,19]. Among the flavonoids, hydroxysafflor yellow A (HSYA) is the main component for the curative effect and chosen as an active marker component for controlling the quality of safflower in Chinese Pharmacopoeia [20]. 

The combination of NS and SF (CNS) could enhance their protective effects against myocardial ischemic injury in our previous studies [10]. The synergistic effects of herbal combination can be achieved by improving the pharmacokinetic profile, including absorption, distribution, metabolism, and excretion [21,22,23,24,25]. Notoginseng saponins and flavonoid glycosides exhibit low oral bioavailability and are eliminated from the body mainly through renal and fecal excretion [26,27]. Our preliminary experiment demonstrated that NS and SF had mutual influence on their respective excretion. Thus, studying the renal and fecal excretion of the active compounds in the combination of NS and SF would aid the investigation of the compatibility mechanism between safflower and notoginseng. 

In the present study, a selective and sensitive LC–MS/MS method was developed and validated for the simultaneous determination of six bioactive constituents (HSYA, notoginsenoide R1, ginsenoside Rb1, Re, Rd, and Rg1) of safflower and notoginseng. The method was applied to the renal and fecal excretion study in rats. The excretions of bioactive constituents were comparatively analyzed following oral administration of NS, SF, and CNS in rats.

## 2. Materials and Methods

### 2.1. Chemicals

NS was purchased from Yunnan Plant Pharmaceutical Co., Ltd. (Kunming, China), and the contents of notoginsenoside R1, ginsenoside Rg1, Re, Rb1, and Rd were 6.2%, 26.6%, 4.1%, 32.5%, and 6.6%, respectively. The quality standard of NS was adopted in accordance with Chinese pharmacopoeia. Safflower was collected from Xinjiang Uygur Autonomous Region (China), and authenticated by Prof. Pengfei Tu. The voucher specimen of safflower (No. 20110301) was deposited at Modern Research Center for Traditional Chinese Medicine, Peking University (Beijing, China). SF was prepared following the protocol reported by Meng, in which the content of HSYA was 10.53% [10]. The ratio of NS and SF was 6:5 in CNS.

HSYA, notoginsenoide R1 and tenuifolin (internal standard, IS) were purchased from Chengdu Must Bio-technology Co., Ltd. (Chengdu, Sichuan, China). Ginsenoside Rb1, Re, Rd, and Rg1 were supplied by National Institutes for Food and Drug Control (Beijing, China). Methanol, acetonitrile, and formic acid (Fisher Scientific, Hampton, NH, USA) were of LC–MS grade. Deionized water was prepared by a Milli-Q water purification system from Millipore (Billerica, MA, USA). 

### 2.2. Animals

Male Sprague Dawley rats (200 ± 20 g) were supplied by the Department of Experimental Animals, Peking University (Beijing, China, animal certificate number: SCXK (Beijing) 2016-0010). The animal room was set at constant temperature of 20 ± 2 °C with humidity of 50 ± 20% and 12/12 h light/dark cycle. The animal studies were approved by the Animal Ethics Committee of Beijing University (No. LA2015061, 27 February 2015), and carried out in accordance with the requirements of China national legislation. 

### 2.3. Excretion Experiments

Eighteen male rats were fasted for 12 h before starting the experiment. The rats were divided into three groups at random (*n* = 6) and received NS (60 mg/kg), SF (50 mg/kg), and NS (60 mg/kg) + SF (50 mg/kg), respectively, by oral gavage. The rats were placed in separate metabolic cages. The urine samples were collected at 0–2, 2–4, 4–6, 6–8, 8–12, 12–24, 24–36, 36–48, 48–72, 72–96, and 96–120 h. The feces samples were collected at 0–4, 4–8, 8–12, 12–24, 24–36, 36–48, 48–72, 72–96, and 96–120 h. The urine volume was recorded and the dry feces were measured for each collection period. Samples were stored at −80 °C until analysis.

### 2.4. Preparation of Calibration Standard and Quality Control (QC) Samples

The standard stock solutions of HSYA, notoginsenoide R1, ginsenoside Rb1, Re, Rd, and Rg1 were prepared by dissolving in methanol with final concentration of 1.00 mg/mL. Then, appropriate aliquots of the six stock solutions were mixed to prepare a final mixed standard solution. The stock solution was diluted with methanol to achieve serial working solutions. The internal standard stock solution was diluted to a concentration of 2 μg/mL with methanol as working solution.

Calibration standard solutions were prepared by spiking 10 μL of working standard solutions with 190 μL blank rat urine (or blank feces water extraction solution) to give nominal concentrations of 10, 20, 50, 100, 200, 500, 1000, 2000, and 5000 ng/mL for HSYA and ginsenoside Rg1, 1, 2, 5, 10, 20, 50, 100, 200, 500, and 1000 ng/mL for notoginsenoide R1, ginsenoside Rb1, Re, and Rd (or 2, 5, 10, 20, 50, 100, 200, 500, 1000, 2000, and 5000 ng/mL for HSYA, notoginsenoide R1, ginsenoside Rg1, Rb1, Re, and Rd). The low, medium, and high concentration levels of the standard solution containing HSYA and ginsenoside Rg1 (10, 200, and 5000 ng/mL), notoginsenoide R1, ginsenoside Rb1, Rd, and Re (1, 20, and 500 ng/mL) for urine and the low, medium, and high concentration levels of the standard solution containing 5, 200, and 2000 ng/mL for feces.

### 2.5. Sample Preparation

An aliquot of 200 μL urine sample was mixed with 10 μL of IS and evaporated to dryness with a ZLS-2 vacuum centrifugal concentrator (Herexi Co., Ltd., Changsha, Hunan, China). The residue was reconstituted in 100 μL of 50% methanol vortexed for 2 min and centrifuged at 12,000 rpm for 15 min. Finally, a 1 μL aliquot was injected for LC–MS analysis.

Feces samples were homogenized in ultrapure water (1 g/15 mL), soaked for 30 min, ultrasonically extracted for 30 min and centrifuged at 9000 rpm for 8 min. An aliquot of 200 μL feces water extraction solution was mixed with 10 μL of IS and then extracted three times with water-saturated *n*-BuOH (150 μL). The upper organic phase was combined and evaporated to dryness using a ZLS-2 vacuum centrifugal concentrator. The residue was treated as the urine sample and a 1-μL aliquot was injected for LC–MS analysis.

### 2.6. LC–MS/MS Analysis

Ultra fast liquid chromatography (UFLC) analysis was performed using a Shimadzu UFLC system (DGU-20A_3R_ Degasser; LC-20AD pump; SIL-20A_XR_ Autosampler; CTO-20AC Column Oven) (Nakagyo-Ku, Koyoto, Japan). The chromatographic separation was achieved with a Waters HSS T3 column (1.8 μm, 2.1 × 50 mm) and the flow rate was 0.4 mL/min. Because of the different endogenous substances in the urine and feces samples, different elution gradients were used to alleviate the matrix effects for the analytes. For urine samples, a gradient elution program was used with mobile phase A (0.01% formic acid in water) and mobile phase B (0.01% formic acid in acetonitrile) as follows 0–4 min, 0–18% B; 4–8 min, 18–54% B; 8–10 min, 54–100% B; 10–11 min, 100% B; 11.1–12 min, and 0% B. For feces samples, a gradient elution program was used with mobile phase A (0.05% formic acid in water) and mobile phase B (0.05% formic acid in acetonitrile) as follows 0–1.5 min, 0–15% B; 1.5–7 min, 15–55% B; 7–8 min, 55–100% B; 8–9 min, 100% B; 9.01–10 min, and 0% B. 

The mass spectrometric detection was performed on an ABSciex 4500 Qtrap mass spectrometer (ABSciex, Foster City, CA, USA) equipped with a negative electrospray ionization (ESI) interface using multiple reaction monitoring (MRM). The parent/fragment ion pairs for each analyte are listed in Table 1. The MS parameters were optimized under the selected ion monitoring conditions as follows: curtain gas, 35 psi; ionspray voltage, 4500 V; turbo gas temperature, 450 °C; GS1, 45 psi; GS2, 45 psi. All data were acquired and analyzed by Analyst software (Versions 1.6.2) from ABSciex.

### 2.7. LC–MS/MS Method Validation

The method was validated for specificity, linearity, the lower limit of quantification (LLOQ), precision and accuracy, recovery, matrix effect, and sample stability. Specificity was assessed by monitoring any endogenous interference in biological samples obtained from six different sources at the retention times of analytes and IS. The calibration curves were assessed by a linear regression with 1/*x*^2^ weighting after plotting the ratios of peak areas of analytes to IS versus corresponding concentration of three independently prepared standard curves. Calibration curves had to have correlation coefficients (*r*) of 0.99 or higher. The limit of detection (LOD) and LLOQ of each analyte were determined based on the signal-to-noise ratio (S/N) of 3 and 10, respectively.

QC samples in five replicates of each analyte were prepared and analyzed on the same day and on three consecutive days to determine the intra- and interday precision and accuracy. The precision was expressed as the relative standard deviation (RSD) between the measured values and the targeted values and were required to be within ±15%, and the accuracy was obtained by calculating the relative error (RE), which was set to be less than ±15% except for LLOQ that should be less than 20%. The extraction recoveries of the analytes were calculated by the ratio between the peak areas of QC samples and that of postextraction spiked blank biological matrix. The matrix effect was investigated by comparing the peak areas of the analytes dissolved in the postextraction blank biological matrix with that of the standard solutions containing equivalent amounts of the analytes.

The sample stability under the experimental conditions was evaluated at low, medium and high levels of QC. The conditions were listed as follows (a) room temperature for 24 h, (b) postpreparation for 96 h at 4 °C, (c) three cycles of freeze and thaw (freezing at −80 °C for 24 h and thawing at room temperature), and (d) long-term storage at −80 °C for 30 days.

### 2.8. Data and Statistical Analysis

The accumulative excretion percentage was calculated for each analyte as follows. Excretion at time (t) = [concentration of the analyte (ng/mL) at t] × total urine (or feces) volume (mL) at t. The cumulative excretion up to time t (mg) was expressed as a percent (%) cumulative excretion by dividing by the total amount of the analyte ingested (mg): % Cumulative Excretion = 100 × (Σ_t_ excretion_t_) total ingested.

All values were expressed as mean ±SD (standard deviation). The unpaired *t*-test was used for statistical analysis of the pharmacokinetic parameters and the value of *p* < 0.05 was considered statistically significant.

## 3. Results and Discussion

### 3.1. Method Validation

#### 3.1.1. Specificity

As shown in Figures S1 and S2 (see Supplementary Materials), comparison of blank biological matrix, blank biological matrix spiked with analytes and IS, and a biological sample after oral administration of CNS indicated no obvious interference at the retention times of the analyte and IS. 

#### 3.1.2. Calibration Curve and Sensitivity

The calibration curves of the analytes exhibited linearity with correlation coefficient (*r*) greater than 0.99 at the tested concentration ranges. The calibration curves, LLOQ of the six analytes are shown in Table 2. 

#### 3.1.3. Precision and Accuracy

Table 3 and Table 4 showed the precision values were within 13.8% for intraday and 14.5% for interday, respectively. The accuracy values ranged from −14.9 to 14.9% in all QC levels. All the values were within the acceptable range. 

#### 3.1.4. Matrix Effect and Extraction Recovery

The extraction recoveries and matrix effect of the six compounds are listed in Table 5. For the analytes at the low, medium, and high concentration levels, the mean extraction recoveries of the analytes were found to be 84.6 to 104.6% and the matrix effects were in range of 88.6 to 109.3%, suggesting that under these LC–MS/MS conditions the analytes had no significant ion suppression or enhancement.

#### 3.1.5. Stability

The stability of the analytes was investigated with the QC samples in rat urine or feces under manifold storage and processing conditions (*n* = 5). The results were summarized in Tables S1 and S2, which showed that all analytes were stable after being stored under a variety of conditions.

### 3.2. Excretion Study

#### 3.2.1. Urinary Excretion Study

**Figure 1:**
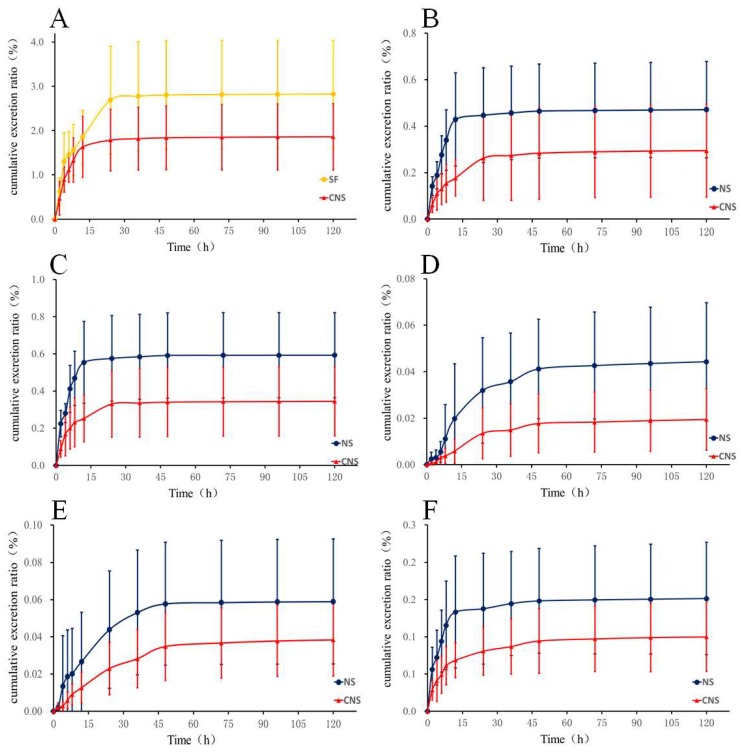
Urinary cumulative excretion profile of (**A**) HSYA in rats after oral administration of SF and CNS and (**B**) Ginsenoside Rg1, (**C**) Ginsenoside R1, (**D**) Ginsenoside Rd, (**E**) Ginsenoside Rb1, and (**F**) Ginsenoside Re in rats after oral administration of NS and CNS (*n* = 6).

The developed LC–MS/MS method was applied to the rat urinary excretion study of HSYA, notoginsenoide R1, ginsenoside Rb1, Re, Rd, and Rg1 following oral administration of NS (60 mg/kg), SF (50 mg/kg), and CNS (110 mg/kg), respectively. The accumulative excreted amounts of the six compounds in urine are shown in Figure 1 and Tables S3–S14. After oral administration of SF and CNS to rats, the cumulative excretion ratios of HSYA were 2.83% and 1.86% within 120 h. The cumulative excretion ratio of HSYA in the CNS group was decreased by 0.66 times comparing with the SF group. In the NS group, the cumulative excretion ratios of notoginsenoide R1, ginsenoside Rb1, Re, Rd, and Rg1 were 0.59%, 0.06%, 0.15%, 0.04%, and 0.47% within 120 h, respectively. Compared with the NS group, the cumulative excretion ratios of the five constituents were decreased by 0.58, 0.67, 0.67, 0.50 (*p* < 0.05), and 0.62 times in the CNS group, respectively. 

#### 3.2.2. Fecal Excretion Study

**Figure 2:**
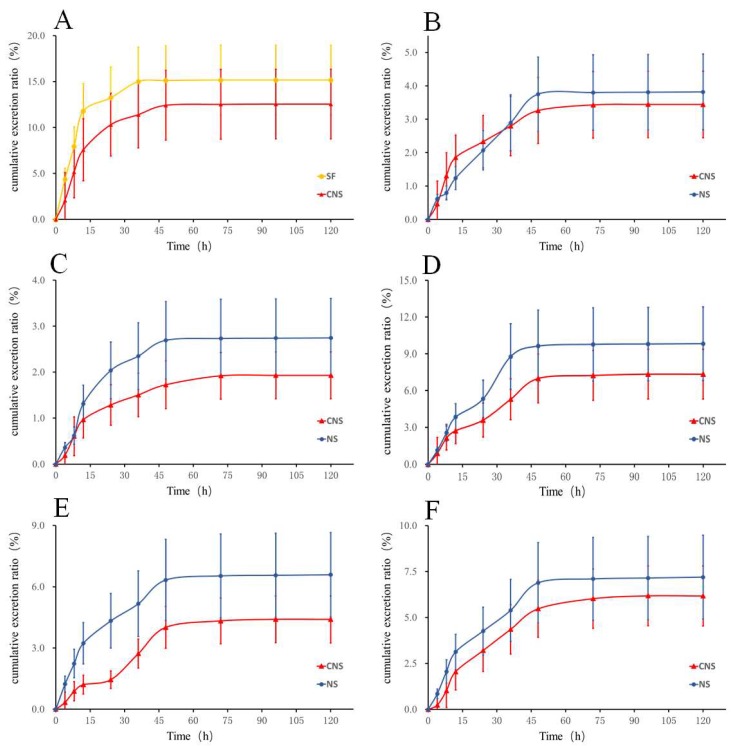
Fecal cumulative excretion profile of (**A**) HSYA in rats after oral administration of SF and CNS and (**B**) Ginsenoside Rg1, (**C**) Ginsenoside R1, (**D**) Ginsenoside Rd, (**E**) Ginsenoside Rb1, and (**F**) Ginsenoside Re in rats after oral administration of NS and CNS (*n* = 6).

As shown in Figure 2 and Tables S15–S26, after oral administration of SF and CNS to rats, the cumulative excretion ratio of HSYA in the CNS group (12.55%) decreased by 0.83 times compared with the SF group (15.16%). In the meantime, the cumulative excretion ratios of notoginsenoide R1, ginsenoside Rb1, Re, Rd, and Rg1 were 9.82%, 2.74%, 7.19%, 6.59%, and 3.82% within 120 h in the NS group, respectively. In the CNS group, the cumulative excretion ratios of the five constituents were decreased by 0.75, 0.70, 0.86, 0.67 (*p* < 0.05), and 0.90 times, respectively, compared with the NS group.

### 3.3. Discussion

We developed and validated a convenient LC–MS/MS method for the simultaneous determination of six bioactive components (hydroxysafflor yellow A (HSYA), notoginsenoside R1, ginsenoside Rb1, Re, Rd, and Rg1) in rat urine and feces after oral administration of notoginseng total saponins (NS), safflower total flavonoids (SF), and the combination of NS and SF (CNS). The total excretion ratios of HSYA, notoginsenoide R1, ginsenoside Rb1, Re, Rd, and Rg1 in the urine and feces were all less than 18% in each group. Moreover, the I-phase metabolism was the major metabolic pathways for the flavonoids in SF in vivo and several saponins in NS could be oxidized by rat liver microsome [25,26]. The results also showed that the combination of safflower and notoginseng could reduce the renal and fecal excretion of six major components after oral administration of CNS comparing with single oral-dosage of SF and NS. It has been reported that HSYA had significant inhibitory effects on CYP1A2 and CYP2C11 in rats. In addition, notoginsenoide R1 was shown to exhibit inhibitory effect on CYP1A2 in rats [28,29]. It is conceivable that the combination of SF and NS could affect the activities of metabolic enzymes and thus the metabolic process of the major constituents of CNS, resulting in lower excretion rates. The lower excretion rates of the major constituents in CNS in urine and feces contributed to the improved efficacy of herbal combination of notogingeng and safflower. 

## 4. Conclusions

A specific, sensitive, and convenient LC–MS/MS method was developed and fully validated for the simultaneous quantitative determination of HSYA, notoginsenoide R1, ginsenoside Rb1, Re, Rd, and Rg1 in rat urine and feces. This method was successfully applied to the study of excretion of six bioactive constituents after oral administration of NS, SF, and CNS, respectively. The combination of safflower and notoginseng reduced the excretion rates of the major flavonoids and saponins in rats compared with the administration of individual. This study elucidated the excretive profile of CNS, SF, and NS in rats and provided the scientific basis for further investigation on the combinatorial use of safflower and notoginseng.

## Figures and Tables

**Table 1:** The parent/fragment ion pairs and MS parameters for the six compounds in CNS and tenuifolin.

Compounds	Q1 (Da)	Q3 (Da)	DP (V)	CE (eV)
HSYA	611.1	491.0	−150	−36
ginsenoside Rg1	845.6	799.5	−85	−38
ginsenoside Rb1	1153.5	1107.4	−103	−37
notoginsenoside R1	977.5	931.5	−98	−30
ginsenoside Rd	991.5	945.5	−100	−31
ginsenoside Re	991.5	945.5	−130	−37
tenuifolin	679.5	455.4	−150	−38

**Table 2:** Liner range, regression equation, and correlation coefficient of six compounds in urine and feces.

Compounds	Matrix	Liner Range (ng/mL)	Regression Equation	Correlation Coefficient (*r*)	LLOQ (ng/mL)	LOD (ng/mL)
HSYA	urine	10–5000	*y* = 0.0023*x* − 0.0011	0.9965	10.0	3.0
	feces	2–5000	*y* = 0.0093x + 0.007	0.9947	2.0	0.6
Ginsenoside Rg1	urine	10–5000	*y* = 0.00092*x* + 0.00057	0.9976	10.0	3.0
	feces	2–5000	*y* = 0.0064*x* + 0.014	0.9950	2.0	0.6
Ginsenoside Rb1	urine	1–1000	*y* = 0.00039*x* − 0.000059	0.9957	1.0	0.3
	feces	2–5000	*y* = 0.00035*x* + 0.00031	0.9909	2.0	0.6
Notoginsenoside R1	urine	1–1000	*y* = 0.0012*x* + 0.000046	0.9950	1.0	0.3
	feces	2–5000	*y* = 0.009*x* + 0.021	0.9977	2.0	0.6
Ginsenoside Rd	urine	1–1000	*y* = 0.0047*x* + 0.00083	0.9903	1.0	0.3
	feces	2–5000	*y* = 0.0068*x* + 0.016	0.9971	2.0	0.6
Ginsenoside Re	urine	1–1000	*y* = 0.00087*x* + 0.00017	0.9969	1.0	0.3
	feces	2–5000	*y* = 0.0056*x* + 0.016	0.9970	2.0	0.6

**Table 3:** Intra-/interday precision and accuracy of six compounds in urine.

Compounds	QC conc. (ng/mL)	Intraday (*n* = 5)			Interday (*n* = 3)		
		Calc. conc (ng/mL)	Precision (%)	Accuracy (%)	Calc. conc (ng/mL)	Precision (%)	Accuracy (%)
HSYA	10	10.2	6.7	1.7	10.2	9.3	2.4
	200	207.6	3.1	3.7	215.7	3.9	7.9
	5000	5550.7	8.6	11.0	5566.0	7.6	11.4
Ginsenoside Rg1	10	9.7	7.4	−2.8	10.0	10.2	−0.7
	200	227.1	2.3	3.5	229.8	2.6	14.9
	5000	5620.8	6.1	12.5	5634.0	6.0	12.9
Ginsenoside Rb1	1	1.0	8.3	1.2	1.0	14.0	−8.5
	20	19.9	8.1	−0.6	21.2	6.4	5.4
	500	507.3	6.7	1.5	505.2	5.5	1.0
Notoginsenoide R1	1	1.0	6.9	−10.3	1.2	14.5	7.2
	20	20.0	6.6	−0.6	20.8	4.9	4.3
	500	502.6	6.3	0.6	487.0	6.6	−2.8
Ginsenoside Rd	1	1.0	13.8	−13.5	1.0	14.0	−1.5
	20	17.4	4.6	−13.3	20.9	5.2	4.4
	500	497.6	7.1	−0.6	449.0	8.4	−10.3
Ginsenoside Re	1	0.9	4.7	−6.6	1.1	10.2	1.3
	20	17.0	9.5	−14.9	19.7	11.6	−2.0
	500	475.4	8.7	−4.9	471.1	6.2	9.8

**Table 4:** Intra-/interday precision and accuracy of six compounds in feces.

Compounds	QC conc. (ng/mL)	Intraday (*n* = 5)			Interday (*n* = 3)		
		Calc. conc (ng/mL)	Precision (%)	Accuracy (%)	Calc. conc (ng/mL)	Precision (%)	Accuracy (%)
HSYA	5	5.0	10.0	6.4	5.4	5.5	6.9
	200	188.5	5.7	−5.9	183.2	5.2	−8.4
	2000	1756.7	2.8	−9.2	1820.0	0.8	−9.0
Ginsenoside Rg1	5	5.4	4.2	7.3	5.4	2.8	8.6
	200	198.0	5.7	−0.9	188.4	5.9	−5.8
	2000	1773.3	3.0	−9.6	1797.5	2.0	−10.1
Ginsenoside Rb1	5	5.4	4.8	8.8	5.4	5.5	7.3
	200	216.5	4.2	8.5	191.7	10.4	−4.1
	2000	2101.7	4.6	5.3	1975.6	7.3	−1.2
Notoginsenoside R1	5	5.4	5.5	7.5	5.3	5.7	6.3
	200	201.2	4.2	0.6	196.4	6.6	−1.8
	2000	1911.7	6.5	−4.5	1813.3	5.8	−9.3
Ginsenoside Rd	5	5.2	8.0	3.0	5.2	8.1	4.0
	200	198.0	4.30	−1.0	191.1	7.3	−4.4
	2000	1920.0	5.64	−4.0	1810.0	5.8	−9.5
Ginsenoside Re	5	5.1	7.8	1.1	5.1	6.5	2.2
	200	199.7	5.8	−0.3	195.5	7.3	−2.2
	2000	1965.0	5.4	−1.6	1882.8	5.1	−5.9

**Table 5:** Recovery and matrix effect of six compounds in urine and feces (*n* = 5).

Compounds	Urine			Feces		
	QC conc. (ng/mL)	Matrix Effect (%)	Recovery (%)	QC conc. (ng/mL)	Matrix Effect (%)	Recovery (%)
HSYA	10	89.9	90.8	5	99.1	92.8
	200	90.4	91.8	200	95.7	91.5
	5000	103.7	101.6	2000	99.9	97.5
Ginsenoside Rg1	10	97.7	96.4	5	88.5	84.6
	200	101.6	101.4	200	94.3	90.9
	5000	93.1	90.2	2000	91.3	90.5
Ginsenoside Rb1	1	104.4	92.5	5	94.1	88.9
	20	109.3	98.2	200	96.3	91.9
	500	93.3	92.0	2000	90.7	89.0
Notoginsenoside R1	1	90.6	96.3	5	93.3	90.6
	20	90.9	90.7	200	103.1	101.9
	500	94.2	91.8	2000	91.0	88.3
Ginsenoside Rd	1	96.8	94.7	5	91.8	87.4
	20	105.9	96.0	200	91.9	90.3
	500	108.0	104.6	2000	90.8	89.6
Ginsenoside Re	1	98.8	93.2	5	91.2	89.6
	20	90.5	91.0	200	104.1	102.5
	500	103.4	100.2	2000	92.0	90.6

## Supplementary Materials

The following are available online at http://www.mdpi.com/1999-4923/10/4/241/s1, Figure S1: Representative LC-MS chromatograms of (a) blank rat urine, (b) rat urine spiked with 6 compounds solution (500 ng/mL) and IS (100 ng/mL), and (c) 6 compounds in the rat urine sample at 0–2 h after oral administration of CNS, Figure S2: Representative LC-MS chromatograms of (a) blank rat feces, (b) rat feces spiked with 6 compounds solution (1000 ng/mL) and IS (100 ng/mL), and (c) 6 compounds in the rat feces sample at 8–12 h after oral administration of CNS, Table S1: Stability of six compounds in rat urine (*n* = 5), Table S2: Stability of six compounds in rat feces (*n* = 5), Table S3: Mean urine excretion amount of HSYA after oral administration of safflower total flavonoids (SF) in rats (*n* = 6, mean ± SD), Table S4: Mean urine excretion amount of HSYA after oral administration of the combination of NS and SF (CNS) in rats (*n* = 6, mean ± SD), Table S5: Mean urine excretion amount of ginsenoside Rg1 after oral administration of notoginseng total saponins (NS) in rats (*n* = 6, mean ± SD), Table S6: Mean urine excretion amount of ginsenoside Rg1 after oral administration of CNS in rats (*n* = 6, mean ± SD), Table S7: Mean urine excretion amount of ginsenoside Rb1 after oral administration of NS in rats (*n* = 6, mean ± SD), Table S8: Mean urine excretion amount of ginsenoside Rb1 after oral administration of CNS in rats (*n* = 6, mean ± SD), Table S9: Mean urine excretion amount of notoginsenoide R1 after oral administration of NS in rats (*n* = 6, mean ± SD), Table S10: Mean urine excretion amount of notoginsenoide R1 after oral administration of CNS in rats (*n* = 6, mean ± SD), Table S11: Mean urine excretion amount of ginsenoside Rd after oral administration of NS in rats (*n* = 6, mean ± SD), Table S12: Mean urine excretion amount of ginsenoside Rd after oral administration of CNS in rats (*n* = 6, mean ± SD), Table S13: Mean urine excretion amount of ginsenoside Re after oral administration of NS in rats (*n* = 6, mean ± SD), Table S14: Mean urine excretion amount of ginsenoside Re after oral administration of CNS in rats (*n* = 6, mean ± SD), Table S15: Mean feces excretion amount of HSYA after oral administration of SF in rats (*n* = 6, mean ± SD), Table S16: Mean feces excretion amount of HSYA after oral administration of CNS in rats (*n* = 6, mean ± SD), Table S17: Mean feces excretion amount of ginsenoside Rg1 after oral administration of NS in rats (*n* = 6, mean ± SD), Table S18: Mean feces excretion amount of ginsenoside Rg1 after oral administration of CNS in rats (*n* = 6, mean ± SD), Table S19: Mean feces excretion amount of ginsenoside Rb1 after oral administration of NS in rats (*n* = 6, mean ± SD), Table S20: Mean feces excretion amount of ginsenoside Rb1 after oral administration of CNS in rats (*n* = 6, mean ± SD), Table S21: Mean feces excretion amount of notoginsenoide R1 after oral administration of NS in rats (*n* = 6, mean ± SD), Table S22: Mean feces excretion amount of notoginsenoide R1 after oral administration of CNS in rats (*n* = 6, mean ± SD), Table S23: Mean feces excretion amount of ginsenoside Rd after oral administration of NS in rats (*n* = 6, mean ± SD), Table S24: Mean feces excretion amount of ginsenoside Rd after oral administration of CNS in rats (*n* = 6, mean ± SD), Table S25: Mean feces excretion amount of ginsenoside Re after oral administration of NS in rats (*n* = 6, mean ± SD), Table S26: Mean feces excretion amount of ginsenoside Re after oral administration of CNS in rats (*n* = 6, mean ± SD).
