# Supplementary material for: Comparative Study on Excretive Characterization of Main Components in Herb Pair Notoginseng-Safflower and Single Herbs by LC–MS/MS
Source: Pharmaceutics. 2018 Nov 18;10(4):241. doi: 10.3390/pharmaceutics10040241 (PMC6321168; doi:10.3390/pharmaceutics10040241)
Supplement: Supplementary file 1 [file pharmaceutics-10-00241-s001.pdf]

pharmaceutics

Supplementary Materials: Comparative Study on Excretive Characterization of Main Components in Herb Pair Notoginseng-Safflower and Single Herbs by LC–MS/MS

Ying-Yuan Lu, Jin-Yang Song, Yan Li, Yu-Qing Meng, Ming-Bo Zhao, Yong Jiang, Peng-Fei Tu and Xiao-Yu Guo

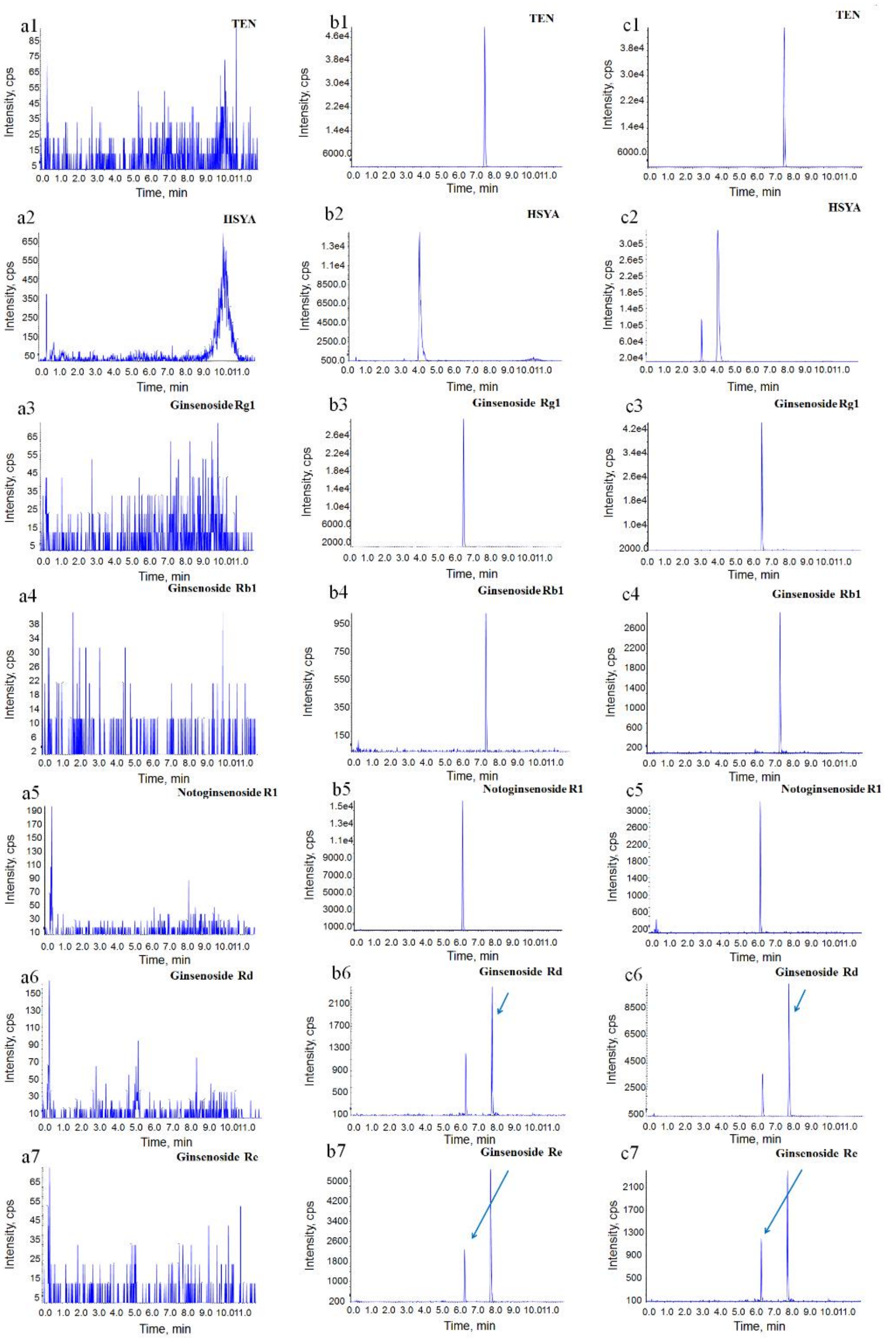

Figure S1. Representative LC-MS chromatograms of (a) blank rat urine, (b) rat urine spiked with 6 compounds solution (500 ng/mL) and IS (100 ng/mL), and (c) 6 compounds in the rat urine sample at 0–2 h after oral administration of CNS.

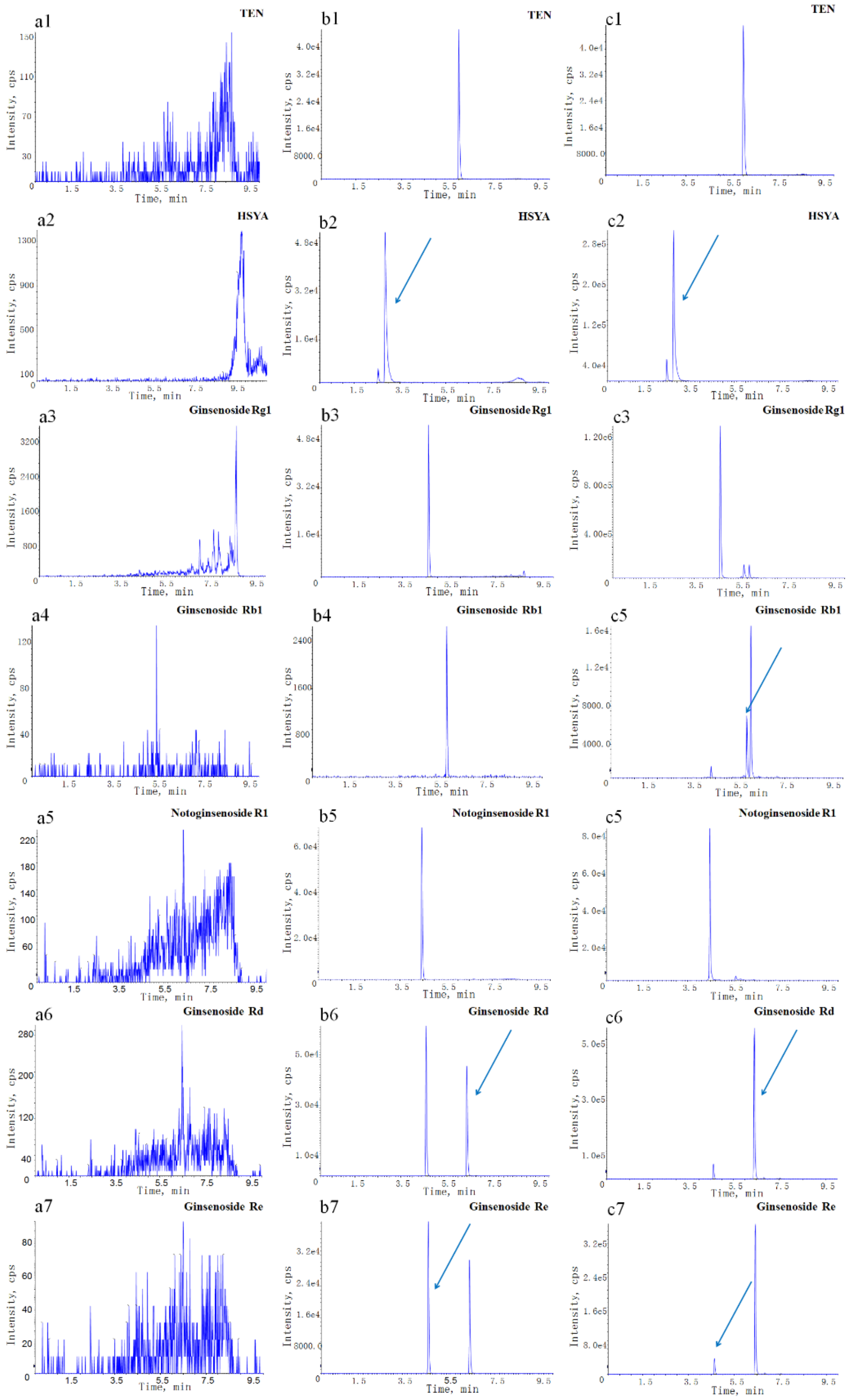

Figure S2. Representative LC-MS chromatograms of (a) blank rat feces, (b) rat feces spiked with 6 compounds solution (1000 ng/mL) and IS (100 ng/mL), and (c) 6 compounds in the rat feces sample at 8–12 h after oral administration of CNS.

Table S1. Stability of six compounds in rat urine (n = 5).

Compounds	QC conc. (ng/mL)	Stored at -80 °C for 1 months			Freeze-thawing 3 cycles			Room temperature for 24 h			Post-preparative stability at 4 °C for 96 h		
		Calc. conc (ng/mL)	Precision (%)	Accuracy (%)	Calc. conc (ng/mL)	Precision (%)	Accuracy (%)	Calc. conc (ng/mL)	Precision (%)	Accuracy (%)	Calc. conc (ng/mL)	Precision (%)	Accuracy (%)
HSYA	10	11.2	8.6	12.5	10.1	10.8	1.5	11.3	9.4	13.2	9.6	13.4	-4.4
	200	173.2	9.4	-13.4	203.0	5.7	1.7	195.8	5.0	-1.9	173.4	10.0	-3.3
	5000	5657.5	8.4	13.2	5104.1	4.9	1.9	5110.0	8.6	2.2	5590.0	5.2	12.0
Ginsenoside Rg1	10	8.8	7.4	12.7	9.6	6.2	-4.3	9.3	10.7	-7.3	9.1	8.2	-8.2
	200	200.9	7.1	0.2	202.3	5.1	1.0	195.7	2.1	-0.4	200.0	4.1	0.1
	5000	5616.0	6.4	12.4	4664.0	3.9	-6.7	5482.1	3.3	9.7	4824.0	3.4	-3.5
Ginsenoside Rb1	1	1.0	7.2	9.2	1.0	7.3	14.9	1.1	10.8	-0.2	0.9	11.6	-5.5
	20	17.6	9.8	-12.5	18.3	7.3	-8.2	17.7	11.2	-11.7	21.6	3.4	8.2
	500	497.5	4.8	-0.6	427.0	2.0	-14.6	452.0	3.5	-9.6	430.0	2.6	-14.1
Notoginsenoide R1	1	1.1	5.2	14.8	0.9	11.0	-13.3	0.9	14.3	-1.1	1.0	14.7	-2.0
	20	17.4	12.4	-7.8	18.7	8.5	-7.0	21.0	10.8	5.0	19.8	7.7	-1.4
	500	501.5	5.4	0.3	436.5	5.8	-12.7	544.0	2.6	8.8	452.2	2.7	-9.5
Ginsenoside Rd	1	1.0	8.4	-6.3	1.1	9.9	5.6	1.0	14.1	-9.2	0.9	14.3	-2.1
	20	17.3	12.7	-13.8	18.3	2.1	-8.7	19.2	12.7	-4.2	19.4	4.7	-3.4
	500	479.1	5.56	-4.3	45.8	5.7	-8.4	473.3	4.1	-5.3	469.6	5.3	-6.2
Ginsenoside Re	1	0.9	13.0	9.7	1.0	9.1	6.2	0.8	14.9	-14.0	0.9	6.5	-13.7
	20	19.5	3.9	-2.3	18.3	3.5	-8.2	10.9	5.8	-3.0	18.1	5.9	-9.0
	500	437.4	5.6	-12.5	465.8	1.4	-6.8	509.0	3.2	1.7	427.3	2.2	-14.6

Table S2. Stability of six compounds in rat feces (*n* = 5).

Compounds	QC conc. (ng/mL)	Stored at -80 °C for 1 months			Freeze-thawing 3 cycles			Room temperature for 24 h			Post-preparative stability at 4 °C for 96 h		
		Calc. conc (ng/mL)	Precision (%)	Accuracy (%)	Calc. conc (ng/mL)	Precision (%)	Accuracy (%)	Calc. conc (ng/mL)	Precision (%)	Accuracy (%)	Calc. conc (ng/mL)	Precision (%)	Accuracy (%)
HSYA	5	4.7	5.9	-6.6	5.0	9.5	0.1	5.0	9.8	1.0	5.4	6.2	6.8
	200	189.3	4.0	-5.3	180.1	6.3	-9.9	178.8	4.1	-7.6	186.8	10.5	-6.4
	2000	2075.0	6.7	4.0	2068.3	4.7	-8.6	1891.7	6.3	-6.8	1783.3	5.0	-10.9
Ginsenoside Rg1	5	5.5	2.1	9.3	5.4	6.1	7.1	5.6	13.2	11.2	5.5	2.5	9.2
	200	214.5	3.1	7.3	203.9	4.2	2.0	217.3	4.1	3.0	212.7	5.4	6.4
	2000	1993.3	1.1	-0.2	1800.0	3.6	2.2	1790.0	3.7	0.4	1891.7	6.2	-5.5
Ginsenoside Rb1	5	5.5	4.5	9.5	5.5	1.2	10.0	4.8	9.7	-4.3	5.4	2.3	8.7
	200	183.3	2.6	-8.4	188.3	5.2	-5.7	186.2	3.3	-3.6	202.5	8.4	1.3
	2000	1903.3	4.4	-4.8	2138.3	3.4	-5.7	1965.0	5.8	-1.7	1881.7	5.8	-6.0
Notoginsenoside R1	5	5.3	4.4	6.2	5.4	3.1	8.7	5.4	4.8	7.3	5.5	2.6	10.5
	200	213.7	2.3	6.8	194.3	4.6	-2.9	213.3	2.7	2.1	214.0	5.0	6.9
	2000	1845.0	5.3	-7.8	1875.0	3.7	-2.1	1808.3	4.1	0.5	1828.3	4.9	-8.5
Ginsenoside Rd	5	5.0	9.4	-0.1	5.1	9.4	2.3	4.8	8.0	-3.8	5.3	5.1	5.4
	200	209.8	2.8	4.8	198.4	2.6	-0.9	213.8	3.9	2.7	209.2	7.6	4.7
	2000	1928.3	2.1	-3.6	1903.3	3.2	-1.4	1826.7	5.2	2.1	1835.0	3.7	-8.2

Ginsenoside Re	5	4.8	9.3	-3.7	5.4	4.5	7.3	5.0	6.2	0.6	5.3	7.0	5.9
	200	219.0	2.6	9.5	192.9	4.0	-3.6	217.2	2.9	4.6	212.8	3.2	6.5
	2000	2095.0	4.4	4.6	1905.0	4.7	-3.3	1866.7	4.8	3.6	1945.0	5.6	-2.6

Table S3. Mean urine excretion amount of HSYA after oral administration of safflower total flavonoids (SF) in rats (n = 6, mean ± SD).

Time point (h)	HSYA Concentration (μg/mL)	Excretion amount (μg)	Accumulative excretion amount (μg)	Accumulative excretion percentage (%)
2	1.803±0.870	6.268±3.141	6.268±3.141	0.623±0.317
4	4.325±2.706	6.880±4.592	13.148±6.505	1.305±0.647
6	3.381±2.681	5.139±5.476	14.717±5.303	1.465±0.527
8	1.310±0.782	2.185±1.420	15.809±5.807	1.564± 0.573
12	0.864±0.046	2.960±2.5891	18.769±6.103	1.861± 0.608
24	0.825±0.828	8.442±8.803	27.211±12.281	2.691±1.210
36	0.201±0.136	0.864±0.722	28.075±12.431	2.782± 1.235
48	0.050±0.046	0.269±0.285	28.344±12.378	2.809±1.228
72	0.022±0.014	0.126±0.043	28.470±12.357	2.822±1.220
96	0.018±0.019	0.031±0.038	28.501±12.334	2.822±1.225
120	0.035±0.045	0.070±0.091	28.562±12.270	2.837±1.216

Table S4. Mean urine excretion amount of HSYA after oral administration of the combination of NS and SF (CNS) in rats (n = 6, mean ± SD).

Time point (h)	HSYA Concentration (μg/mL)	Excretion amount (μg)	Accumulative excretion amount (μg)	Accumulative excretion percentage (%)
2	3.367±3.471	4.713±3.816	4.713±3.816	0.473±0.387
4	4.299±4.350	5.787±2.672	9.054± 2.878	0.902±0.288
6	1.851±1.081	2.915±1.407	11.240±2.799	1.111±0.284
8	2.374±2.051	3.567±3.630	13.469±5.034	1.332±0.503
12	4.148±6.221	3.442±2.613	16.481±6.950	1.634±0.696

24	0.453±0.548	1.562±1.307	18.044±7.031	1.783±0.701
36	0.198±0.296	0.340±0.319	18.373±7.165	1.821±0.715
48	0.039±0.042	0.195±0.178	18.568±7.270	1.841±0.726
72	0.066±0.139	0.133±0.278	18.701±7.480	1.857±0.748
96	0.031±0.045	0.062±0.089	18.763±7.536	1.864±0.759
120	0.057± 0.114	0.041±0.052	18.804±7.544	1.864±0.756

Table S5. Mean urine excretion amount of ginsenoside Rg1 after oral administration of notoginseng total saponins (NS) in rats (n = 6, mean ± SD).

Time point (h)	Ginsenoside Rg1 Concentration (μg/mL)	Excretion amount (μg)	Accumulative excretion amount (μg)	Accumulative excretion percentage (%)
2	3.362±7.021	4.985±1.411	4.985±1.411	0.142±0.045
4	1.678±1.800	2.844±0.973	6.610±2.052	0.193±0.061
6	1.445±1.455	3.616±2.575	9.709±2.893	0.285±0.086
8	1.140±1.226	3.890±2.477	11.931±4.568	0.348±0.137
12	0.727±0.171	3.130±2.715	15.061±7.022	0.439±0.200
24	0.135±0.209	0.636±0.590	15.697±7.182	0.451±0.202
36	0.115±0.151	0.402±0.338	16.042±7.062	0.463±0.204
48	0.100±0.171	0.285±0.276	1.633±7.089	0.465±0.204
72	0.178±0.382	0.091±0.014	16.417±7.147	0.476±0.208
96	0.085±0.159	0.062±0.110	16.480±7.192	0.473±0.208
120	0.059±0.105	0.062±0.148	16.542±7.264	0.473±0.211

Table S6. Mean urine excretion amount of ginsenoside Rg1 after oral administration of CNS in rats (n = 6, mean ± SD).

Time point (h)	Ginsenoside Rg1 Concentration (μg/mL)	Excretion amount (μg)	Accumulative excretion amount (μg)	Accumulative excretion percentage (%)
2	1.507±1.443	1.881±1.001	1.881±1.001	0.066±0.032
4	1.692±2.343	2.002±1.609	3.382±2.188	0.111±0.072
6	0.544±0.216	0.816±0.264	3.994±2.061	0.132±0.073
8	0.835±0.697	1.169±0.913	4.725±2.486	0.155±0.084
12	0.789±0.937	0.805±0.300	5.430±2.374	0.188±0.080
24	2.348±5.832	2.622±5.710	8.052±5.572	0.263±0.182
36	0.228±0.361	0.336±0.421	8.388±5.927	0.274±0.195
48	0.065±0.082	0.301±0.361	8.689±6.056	0.285±0.202
72	0.093±0.156	0.185±0.312	8.87±6.051	0.293±0.206
96	0.046±0.053	0.091±0.106	8.966±6.090	0.292±0.204
120	0.080±0.174	0.061±0.081	9.027±6.117	0.292±0.206

Table S7. Mean urine excretion amount of ginsenoside Rb1 after oral administration of NS in rats (n = 6, mean ± SD).

Time point (h)	Ginsenoside Rb1 Concentration (μg/mL)	Excretion amount (μg)	Accumulative excretion amount (μg)	Accumulative excretion percentage (%)
2	0.115±0.284	0.075±0.063	0.075±0.063	0.002±0.001
4	0.245±0.450	0.872±1.518	0.574±1.167	0.013±0.027
6	0.140±0.244	0.262±0.207	0.798±1.077	0.019±0.025
8	0.140±0.196	0.152±0.084	0.863±1.044	0.021±0.025
12	0.214±0.344	0.276±0.142	1.139±1.140	0.027±0.027

24	0.240±0.553	0.742±0.289	1.881±1.352	0.044±0.032
36	0.061±0.054	0.394±0.221	2.275±1.435	0.053±0.033
48	0.142±0.344	0.201±0.117	2.476±1.415	0.058±0.033
72	0.084±0.195	0.030±0.020	2.506±1.429	0.058±0.033
96	0.045±0.104	0.016±0.011	2.521±1.438	0.060±0.034
120	0.025±0.062	0.006±0.003	2.528±1.439	0.060±0.034

Table S8. Mean urine excretion amount of ginsenoside Rb1 after oral administration of CNS in rats (n = 6, mean ± SD)`.

Time point (h)	Ginsenoside Rb1 Concentration (μg/mL)	Excretion amount (μg)	Accumulative excretion amount (μg)	Accumulative excretion percentage (%)
2	0.054±0.073	0.075±0.093	0.075±0.093	0.002±0.002
4	0.027±0.041	0.044±0.050	0.106±0.086	0.003±0.002
6	0.111±0.121	0.158±0.142	0.218±0.166	0.006±0.004
8	0.105±0.100	0.212±0.169	0.339±0.213	0.009±0.006
12	0.120±0.134	0.154±0.128	0.472±0.310	0.013±0.008
24	0.072 ±0.050	0.388±0.391	0.859±0.527	0.023±0.014
36	0.138±0.144	0.197±0.129	1.056±0.587	0.028±0.016
48	0.051±0.060	0.247±0.216	1.303±0.684	0.035±0.018
72	0.033±0.028	0.070±0.060	1.373±0.705	0.037±0.019
96	0.019±0.014	0.040±0.031	1.414±0.715	0.038±0.019
120	0.014±0.012	0.022±0.025	1.436±0.726	0.038±0.019

Table S9. Mean urine excretion amount of notoginsenoide R1 after oral administration of NS in rats (n = 6, mean ± SD).

Time point (h)	Notoginsenoside R1 Concentration (μg/mL)	Excretion amount (μg)	Accumulative excretion amount (μg)	Accumulative excretion percentage (%)
2	0.347±0.206	1.836±0.582	1.836±0.583	0.221±0.074
4	0.554±0.525	0.923±0.305	2.297±0.417	0.282±0.055
6	0.487±0.591	1.291±1.148	3.373±1.035	0.414±0.133
8	0.303±0.337	0.914±0.716	3.830±1.192	0.476±0.151
12	0.160±0.020	0.703±0.729	4.533±1.801	0.557±0.229
24	0.025±0.032	0.177±0.156	4.710±1.879	0.586±0.237
36	0.017±0.018	0.072±0.047	4.782±1.871	0.582±0.233
48	0.014±0.020	0.057±40.00	4.840±1.877	0.591±0.232
72	0.020±0.038	0.006±0.005	4.846±1.877	0.591±0.235
96	0.009±0.014	0.003±0.003	4.849±1.876	0.591±0.232
120	0.008±0.013	0.001±0.0001	4.850±1.876	0.593±0.231

Table S10. Mean urine excretion amount of notoginsenoide R1 after oral administration of CNS in rats (n = 6, mean ± SD).

Time point (h)	Notoginsenoside R1 Concentration (μg/mL)	Excretion amount (μg)	Accumulative excretion amount (μg)	Accumulative excretion percentage (%)
2	0.499±0.465	0.637±0.317	0.637±0.317	0.091±0.043
4	0.664±1.002	0.750±0.709	1.200±0.836	0.174±0.122
6	0.195±0.094	0.301±0.152	1.426±0.803	0.201±0.115
8	0.264±0.226	0.454±0.236	1.653±0.940	0.231±0.134
12	0.159±0.204	0.177±0.097	1.808±0.888	0.252±0.123

24	0.480±0.130	0.554±0.128	2.361±1.275	0.334±0.182
36	0.021±0.037	0.037±0.053	2.399±1.316	0.345±0.186
48	0.007±0.005	0.033±0.019	2.432±1.326	0.341±0.196
72	0.008±0.011	0.016±0.022	2.447±1.325	0.342±0.193
96	0.004±0.003	0.009±0.007	2.456±1.328	0.342±0.193
120	0.005±0.004	0.006±0.003	2.462±1.328	0.342±0.196

Table S11. Mean urine excretion amount of ginsenoside Rd after oral administration of NS in rats (*n* = 6, mean ± SD).

Time point (h)	Ginsenoside Rd Concentration (μg/mL)	Excretion amount (μg)	Accumulative excretion amount (μg)	Accumulative excretion percentage (%)
2	0.017±0.039	0.022±0.025	0.022±0.025	0.002±0.003
4	0.033±0.069	0.008±0.003	0.027±0.029	0.003±0.003
6	0.010±0.018	0.021±0.020	0.048±0.039	0.006±0.004
8	0.009±0.012	0.082±0.123	0.097±0.129	0.011±0.015
12	0.028±0.019	0.076±0.083	0.173±0.206	0.020±0.024
24	0.034±0.063	0.106±0.109	0.279±0.198	0.032±0.023
36	0.017±0.033	0.041±0.012	0.311±0.182	0.036±0.021
48	0.012±0.019	0.048±0.045	0.359±0.187	0.041±0.021
72	0.012±0.023	0.013±0.018	0.372±0.200	0.043±0.023
96	0.006±0.010	0.008±0.013	0.379±0.211	0.044±0.024
120	0.003±0.006	0.007±0.012	0.386±0.221	0.044±0.025

Table S12. Mean urine excretion amount of ginsenoside Rd after oral administration of CNS in rats (n = 6, mean ± SD).

Time point (h)	Ginsenoside Rd Concentration (μg/mL)	Excretion amount (μg)	Accumulative excretion amount (μg)	Accumulative excretion percentage (%)
2	0.022±0.042	0.005±0.005	0.005±0.005	0.001±0.001
4	0.009±0.015	0.005±0.007	0.009±0.008	0.001±0.001
6	0.017±0.016	0.018±0.018	0.024±0.023	0.003±0.003
8	0.038±0.075	0.010±0.009	0.029±0.024	0.004±0.003
12	0.053±0.110	0.019±0.016	0.045±0.040	0.006±0.005
24	0.315±0.787	0.058±0.066	0.103±0.083	0.014±0.011
36	0.041±0.068	0.011±0.009	0.114±0.086	0.015±0.011
48	0.030±0.070	0.021±0.024	0.135±0.096	0.018±0.013
72	0.016±0.035	0.004±0.004	0.139±0.098	0.018±0.013
96	0.016±0.036	0.005±0.005	0.144±0.100	0.019±0.013
120	0.010±0.017	0.004±0.004	0.148±0.101	0.019±0.013

Table S13. Mean urine excretion amount of ginsenoside Re after oral administration of NS in rats (n = 6, mean ± SD).

Time point (h)	Ginsenoside Re Concentration (μg/mL)	Excretion amount (μg)	Accumulative excretion amount (μg)	Accumulative excretion percentage (%)
2	0.289±0.689	0.304±0.161	0.304±0.161	0.064±0.030
4	0.091±0.119	0.129±0.051	0.390±0.199	0.072±0.037
6	0.032±0.030	0.143±0.125	0.509±0.225	0.096±0.042
8	0.040±0.026	0.173±0.121	0.625±0.321	0.127±0.059
12	0.033±0.024	0.096±0.087	0.721±0.406	0.138±0.075

24	0.017±0.034	0.024±0.015	0.745±0.403	0.149±0.074
36	0.015±0.023	0.044±0.046	0.782±0.377	0.141±0.070
48	0.013±0.024	0.020±0.022	0.802±0.380	0.152±0.070
72	0.017±0.037	0.007±0.017	0.809±0.391	0.156±0.072
96	0.007±0.013	0.005±0.010	0.814±0.399	0.154±0.074
120	0.004±0.008	0.004±0.010	0.819±0.408	0.154±0.075

Table S14. Mean urine excretion amount of ginsenoside Re after oral administration of CNS in rats (*n* = 6, mean ± SD).

Time point (h)	Ginsenoside Re Concentration (μg/mL)	Excretion amount (μg)	Accumulative excretion amount (μg)	Accumulative excretion percentage (%)
2	0.086±0.088	0.133±0.062	0.133±0.062	0.028±0.013
4	0.070±0.089	0.100±0.090	0.193±0.131	0.041±0.028
6	0.039±0.027	0.064±0.030	0.231±0.118	0.049± 0.025
8	0.041±0.035	0.072±0.033	0.289±0.121	0.061±0.026
12	0.035±0.041	0.035±0.015	0.324±0.110	0.069±0.023
24	0.369±0.963	0.058±0.054	0.381±0.153	0.081±0.032
36	0.024±0.038	0.030±0.037	0.411±0.177	0.087±0.037
48	0.006±0.007	0.03±0.038	0.447±0.206	0.095±0.044
72	0.005±0.007	0.013±0.017	0.460±0.207	0.097±0.044
96	0.003±0.005	0.008±0.012	0.468±0.217	0.099±0.046
120	0.004±0.008	0.004±0.005	0.472±0.219	0.100±0.046

Table S15. Mean feces excretion amount of HSYA after oral administration of SF in rats (n = 6, mean ± SD).

Time point (h)	HSYA Concentration (μg/mL)	Excretion amount (μg)	Accumulative excretion amount (μg)	Accumulative excretion percentage (%)
4	2.409±0.352	43.994±12.319	43.994±12.319	4.352±1.224
8	1.954±0.115	36.072±10.358	80.066±21.692	7.922±2.151
12	2.144±0.142	39.052±8.681	119.118±30.243	11.784±2.995
24	0.791±0.082	14.632±4.256	133.750±33.764	13.233±3.346
36	0.975±0.039	17.848±4.279	151.598±37.989	15.001±3.762
48	0.060±0.002	1.109±0.297	152.707±38.286	15.115±3.794
72	0.031±0.002	0.570±0.162	153.277±38.442	15.161±3.803
96	ND	ND	153.277±38.442	15.156±3.807
120	ND	ND	153.277±38.442	15.159±3.802

Table S16. Mean feces excretion amount of HSYA after oral administration of CNS in rats (n = 6, mean ± SD).

Time point (h)	HSYA Concentration (μg/mL)	Excretion amount (μg)	Accumulative excretion amount (μg)	Accumulative excretion percentage (%)
4	1.646±2.261	21.013±30.299	21.012±30.299	2.084±3.005
8	2.095±0.121	31.265±8.953	52.277±28.669	5.171±2.844
12	1.660±0.349	24.260±6.796	76.537±34.048	7.572±3.374
24	1.829±0.220	27.706±9.755	104.243±34.486	10.316±3.415
36	0.737±0.037	11.040±3.298	115.284±36.570	11.401±3.623
48	0.688±0.047	10.267±3.026	125.551±38.391	12.422±3.804
72	0.068±0.028	1.049±0.586	126.560±38.340	12.521±3.795

96	0.017±0.016	0.274±0.283	126.874±38.274	12.556±3.795
120	ND	ND	126.874±38.274	12.556±3.796

Table S17. Mean feces excretion amount of ginsenoside Rg1 after oral administration of NS in rats (*n* = 6, mean ± SD).

Time point (h)	Ginsenoside Rg1 Concentration (μg/mL)	Excretion amount (μg)	Accumulative excretion amount (μg)	Accumulative excretion percentage (%)
4	1.508±0.256	21.063±5.410	21.063±5.410	0.612±0.145
8	0.473±0.016	6.792±2.122	27.855±7.375	0.798±0.216
12	1.087±0.088	15.587±4.879	43.442.±12.025	1.244±0.343
24	2.041±0.055	29.239±8.754	72.682±20.662	2.071±0.592
36	2.020±0.054	28.977±8.942	101.659±29.524	2.904±0.845
48	2.084±0.047	30.041±9.594	131.700±39.092	3.751±1.111
72	0.116±0.008	1.673±0.554	133.372±39.629	3.801±1.132
96	0.023±0.001	0.327±0.104	133.700±39.733	3.814±1.133
120	0.022±0.002	0.315±0.110	134.015±39.837	3.824±1.132

Table S18. Mean feces excretion amount of ginsenoside Rg1 after oral administration of CNS in rats (*n* = 6, mean ± SD).

Time point (h)	Ginsenoside Rg1 Concentration (μg/mL)	Excretion amount (μg)	Accumulative excretion amount (μg)	Accumulative excretion percentage (%)
4	1.105±1.515	14.286±20.907	14.286±20.907	0.474±0.681
8	1.708±0.054	25.666.±7.933	39.952±21.244	1.303±0.695
12	1.109±0.221	16.908±6.636	56.860±20.559	1.864±0.675
24	0.980±0.138	14.504±4.143	71.363±24.096	2.331±0.796

36	0.959±0.040	14.365±4.335	85.729±27.211	2.802±0.894
48	0.943±0.049	14.173±4.533	99.901±30.374	3.265±0.994
72	0.324±0.249	5.212±4.367	105.113±30.466	3.431±0.996
96	0.024±0.024	0.396±0.402	105.509±30.482	3.441±0.996
120	ND	ND	105.509±30.482	3.442±0.994

Table S19. Mean feces excretion amount of ginsenoside Rb1 after oral administration of NS in rats (*n* = 6, mean ± SD).

Time point (h)	Ginsenoside Rb1 Concentration (μg/mL)	Excretion amount (μg)	Accumulative excretion amount (μg)	Accumulative excretion percentage (%)
4	1.082± 0.156	15.357±4.697	15.357±4.697	0.361±0.113
8	0.767±0.029	11.066±3.551	26.423±8.057	0.624±0.192
12	2.064±0.137	29.724±9.595	56.148±17.467	1.312±0.411
24	2.184±0.090	31.223±9.132	87.371±26.505	2.043±0.622
36	0.920±0.048	13.350±4.715	100.721±31.049	2.354±0.725
48	1.022±0.052	14.806±5.076	115.527±36.105	2.692±0.846
72	0.120±0.009	1.710±0.525	117.237±36.600	2.731±0.854
96	0.019±0.004	0.264±0.103	117.502±36.686	2.741±0.854
120	0.017±0.003	0.252±0.100	117.753±36.774	2.745±0.858

Table S20. Mean feces excretion amount of ginsenoside Rb1 after oral administration of CNS in rats (*n* = 6, mean ± SD).

Time point (h)	Ginsenoside Rb1 Concentration (μg/mL)	Excretion amount (μg)	Accumulative excretion amount (μg)	Accumulative excretion percentage (%)
4	0.555±0.763	7.072±10.199	7.072±10.199	0.192±0.275

8	1.079±0.431	15.499±6.228	22.570±15.790	0.607±0.426
12	0.899±0.096	13.492±4.335	36.062±14.935	0.968±0.409
24	0.802±0.025	12.018±3.497	48.081±16.475	1.282±0.443
36	0.548±0.036	8.141±2.259	56.222±17.772	1.501±0.476
48	0.552±0.030	8.316±2.614	64.538±19.434	1.723±0.522
72	0.455±0.377	7.325±6.432	71.863±19.005	1.923±0.515
96	0.021±0.020	0.350±0.350	72.213±19.048	1.931±0.512
120	ND	ND	72.213±19.048	1.931±0.513

Table S21. Mean feces excretion amount of notoginsenoide R1 after oral administration of NS in rats (*n* = 6, mean ± SD).

**Time point (h)**	**Notoginsenoside R1 Concentration (μg/mL)**	**Excretion amount (μg)**	**Accumulative excretion amount (μg)**	**Accumulative excretion percentage (%)**
4	0.654±0.107	9.116±2.220	9.116±2.220	1.112±0.274
8	0.819±0.021	11.769±3.631	20.885±5.710	2.552±0.705
12	0.734±0.058	10.516±3.227	31.400±8.858	3.843±1.083
24	0.852±0.028	12.201±3.654	43.602±12.487	5.331±1.532
36	1.949±0.092	28.179±9.578	71.781±21.933	8.772±2.685
48	0.486±0.014	7.000±2.190	78.781±24.115	9.637±2.956
72	0.081±0.006	1.167±0.367	79.948±24.475	9.773±2.992
96	0.015±0.001	0.222±0.076	80.170±24.552	9.806±3.001
120	0.015±0.002	0.212±0.077	80.382±24.620	9.826±3.017

Table S22. Mean feces excretion amount of notoginsenoide R1 after oral administration of CNS in rats (*n* = 6, mean ± SD).

Time point (h)	Notoginsenoside R1 Concentration (μg/mL)	Excretion amount (μg)	Accumulative excretion amount (μg)	Accumulative excretion percentage (%)
4	0.498±0.687	6.325±9.106	6.325±9.106	0.891±1.272
8	0.579±0.146	8.872±3.877	15.196±6.994	2.135±0.983
12	0.286±0.016	4.255±1.140	19.451±7.478	2.726±1.057
24	0.430±0.169	6.1.98±2.470	25.649±9.940	3.594±1.392
36	0.817±0.034	12.178±3.423	37.827±11.858	5.305±1.667
48	0.807±0.056	12.106±3.723	49.933±14.254	6.992±2.001
72	0.116±0.021	1.766±0.672	51.699±14.529	7.243±2.035
96	0.044±0.042	0.713±0.707	52.411±14.487	7.342±2.034
120	ND	ND	52.411±14.487	7.342± 2.033

Table S23. Mean feces excretion amount of ginsenoside Rd after oral administration of NS in rats (*n* = 6, mean ± SD).

Time point (h)	Ginsenoside Rd Concentration (μg/mL)	Excretion amount (μg)	Accumulative excretion amount (μg)	Accumulative excretion percentage (%)
4	0.752±0.079	10.755±3.385	10.755±3.385	1.232±0.395
8	0.605±0.022	8.721±2.813	19.476±6.086	2.244± 0.706
12	0.602±0.049	8.676±2.808	28.152±8.861	3.232±1.024
24	0.670±0.034	9.562±2.795	37.713±11.637	4.331±1.342
36	0.505±0.014	7.290±2.427	45.004±14.004	5.174±1.612
48	0.704±0.026	10.151±3.361	55.154±17.338	6.332±1.991
72	0.122±0.005	1.753±0.562	56.907±17.892	6.534±2.048

96	0.017±0.001	0.241±0.076	57.148±17.967	6.563±2.059
120	0.015±0.002	0.223±0.076	57.371±18.037	6.589±2.068

Table S24. Mean feces excretion amount of ginsenoside Rd after oral administration of CNS in rats (*n* = 6, mean ± SD).

Time point (h)	Ginsenoside Rd Concentration (μg/mL)	Excretion amount (μg)	Accumulative excretion amount (μg)	Accumulative excretion percentage (%)
4	0.204±0.283	2.579±3.701	2.579±3.701	0.343±0.491
8	0.274±0.065	4.108±1.195	6.687±3.535	0.883±0.462
12	0.164±0.012	2.459±0.753	9.145±3.524	1.204±0.465
24	0.119±0.051	1.855±1.046	11.000±3.256	1.453±0.432
36	0.653±0.041	9.700±2.660	20.700±5.415	2.724±0.715
48	0.658±0.072	9.778±2.743	30.478±7.801	4.011±1.032
72	0.153±0.072	2.403±1.477	32.881±8.515	4.322±1.123
96	0.035±0.034	0.570±0.568	33.451±8.722	4.403±1.154
120	ND	ND	33.425±8.713	4.404±1.151

Table S25. Mean feces excretion amount of ginsenoside Re after oral administration of NS in rats (*n* = 6, mean ± SD).

Time point (h)	Ginsenoside Re Concentration (μg/mL)	Excretion amount (μg)	Accumulative excretion amount (μg)	Accumulative excretion percentage (%)
4	0.323±0.043	4.585±1.408	4.585±1.408	0.852±0.261
8	0.455±0.018	6.563±2.122	11.148±3.459	2.063±0.644
12	0.401±0.027	5.759±1.787	16.907±5.229	3.122±0.971
24	0.430±0.015	6.150±1.850	23.057±7.062	4.266±1.305

36	0.421±0.018	6.105±2.098	29.162±9.141	5.397± 1.699
48	0.561±0.026	8.106±2.700	37.268±11.835	6.892±2.193
72	0.081±0.006	1.155±0.306	38.423±12.180	7.103±2.254
96	0.018±0.001	0.262±0.086	38.685±12.265	7.149±2.268
120	0.017±0.002	0.252±0.091	38.937±12.350	7.187±2.278

Table S26. Mean feces excretion amount of ginsenoside Re after oral administration of CNS in rats (*n* = 6, mean ± SD).

Time point (h)	Ginsenoside Re Concentration (μg/mL)	Excretion amount (μg)	Accumulative excretion amount (μg)	Accumulative excretion percentage (%)
4	0.087±0.124	1.098±1.616	1.098±1.616	0.234±0.345
8	0.267±0.204	3.705±2.813	4.802±4.393	1.022±0.936
12	0.330±0.026	4.873±1.226	9.675±4.726	2.051±1.001
24	0.366±0.020	5.459±1.584	15.135±5.426	3.203±1.152
36	0.367±0.026	5.435±1.413	20.570±6.347	4.364±1.343
48	0.354±0.028	5.282±1.513	25.852±7.369	5.469±1.568
72	0.168±0.069	2.615±1.424	28.467±7.628	6.027±1.613
96	0.044±0.043	0.717±0.713	29.184±7.675	6.182±1.623
120	ND	ND	29.184±7.675	6.182±1.623
